# Genetic-based dissection of resistance to bacterial leaf streak in rice by GWAS

**DOI:** 10.1186/s12870-023-04412-7

**Published:** 2023-08-18

**Authors:** Xiaoyang Zhu, Lei Chen, Zhanying Zhang, Jinjie Li, Hongliang Zhang, Zichao Li, Yinghua Pan, Xueqiang Wang

**Affiliations:** 1 State Key Laboratory of Agrobiotechnology / Beijing Key Laboratory of Crop Genetic Improvement, China Agricultural University, Beijing, 100193 China; 2 Hainan Yazhou Bay Seed Laboratory, Sanya, Hainan 572025 People’s Republic of China; 3 Guangxi Key Laboratory of Rice Genetics and Breeding, Rice Research Institute, Guangxi Academy of Agricultural Sciences, Nanning, 530007 China; 4 Zhejiang Provincial Key Laboratory of Crop Genetic Resources, College of Agriculture and Biotechnology, Zhejiang University, Hangzhou, 310058 China

**Keywords:** Genome-wide association study, Rice, Bacterial leaf streak, Haplotype analysis, Transcriptome analysis

## Abstract

**Background:**

Rice is the second-largest food crop in the world and vulnerable to bacterial leaf streak disease. A thorough comprehension of the genetic foundation of agronomic traits was essential for effective implementation of molecular marker-assisted selection.

**Results:**

Our study aimed to evaluate the vulnerability of rice to bacterial leaf streak disease (BLS) induced by the gram-negative bacterium *Xanthomonas oryzae* pv. *oryzicola* (Xoc). In order to accomplish this, we first analyzed the population structure of 747 accessions and subsequently assessed their phenotypes 20 days after inoculation with a strain of Xoc, GX01. We conducted genome-wide association studies (GWAS) on a population of 747 rice accessions, consisting of both *indica* and *japonica* subpopulations, utilizing phenotypic data on resistance to bacterial leaf streak (RBLS) and sequence data. We identified a total of 20 QTLs associated with RBLS in our analysis. Through the integration of linkage mapping, sequence analysis, haplotype analysis, and transcriptome analysis, we were able to identify five potential candidate genes (*OsRBLS1*—*OsRBLS5*) that possess the potential to regulate RBLS in rice. In order to gain a more comprehensive understanding of the genetic mechanism behind resistance to bacterial leaf streak, we conducted tests on these genes in both the *indica* and *japonica* subpopulations, ultimately identifying superior haplotypes that suggest the potential utilization of these genes in breeding disease-resistant rice varieties.

**Conclusions:**

The findings of our study broaden our comprehension of the genetic mechanisms underlying RBLS in rice and offer significant insights that can be applied towards genetic improvement and breeding of disease-resistant rice in rapidly evolving environmental conditions.

**Supplementary Information:**

The online version contains supplementary material available at 10.1186/s12870-023-04412-7.

## Background

Rice, which is considered as the representative species of the Oryza genus, is a cereal crop that originated in China and India. It has been cultivated for over 7,000 years in the Yangtze River basin of China and has played a fundamental role in the development of human civilization [1, 2]. Rice was susceptible to bacterial leaf streak disease (BLS), which was caused by the gram-negative bacterium *Xanthomonas oryzae* pv. *oryzicola* (Xoc), a pathogen that specifically targets rice [3, 4]. BLS was a major bacterial disease that can severely impact rice production and had been classified as an important quarantine disease in China [4–6]. The major factors that contributed to the onset of BLS primarily consisted of pathogenic bacteria with virulent properties, susceptible host plants, and favorable conditions that promoted disease development [7]. The geographic range of BLS had gradually increased due to the widespread distribution of hybrid rice seeds and southern-bred rice varieties. BLS could lead to yield losses that range from 40 to 60%, thereby considerably affecting rice production [8, 9]. While no naturally immune rice varieties had been discovered in nature, the identification and exploration of BLS-resistant genes can help in the development of new high-resistance rice varieties to diminish the negative impact of BLS on rice yield and quality. In general, this approach can lead to a scientific, effective, and rational means of controlling BLS.

The initial step towards identifying resistance genes and developing resistant rice varieties was the precise characterization of resistance to bacterial leaf streak (BLS) in rice. Here, they evaluated four different inoculation methods for rice BLS disease (namely, spraying, leaf cutting, acupuncture, and infiltration) across 10 diverse rice accessions. The results indicated that both acupuncture and infiltration methods were effective in differentiating the varying degrees of disease resistance or susceptibility among the 10 rice accessions [10]. Few studies had explored BLS resistance genes, and the molecular mechanisms underlying rice resistance to Xoc and the pathogenicity of this bacterium were not well understood. BC_2_ generation using the varieties H359 and Jiannong 8 revealed that resistance to BLS was a quantitative trait controlled by multiple genes [11]. Identified quantitative traits controlled by multiple genes were shown to explain the genetic mode of BLS resistance [12]. Using composite interval mapping, five QTLs with significant contributions were identified in the resistant population [13]. Using the BLS-resistant near-isogenic line H359R, the genomic composition of resistance QTLs was analyzed by genotype display analysis. The results showed that the BLS-resistant parent Acc8558 contained three resistance QTL regions, while the susceptible parent H359 contained only one resistance QTL region [14]. One QTL, *qBlsr5a*, was subsequently mapped to 30 kb using a sub-chromosome segment substitution line, suggesting that the most likely candidate gene was LOC_Os05g01710 [15]. A mapping population was constructed by crossing wild rice *Oryza rufipogon Griff* resistant source 'DY19' with *indica* rice variety 9311. The results showed that 'DY19' was controlled by a pair of new major recessive genes, *bls2*, located between SL03 and SL04 on chromosome 2, within a range of about 4 cM [16]. A mapping and separation population was constructed using the highly resistant international rice variety BJ1 and the highly susceptible local high-quality rice variety Youzhan 8 as parents. A recessive BLS-resistant gene was identified in BJ1 and this gene mapped to chromosome 10 at about 48.8 cM and was closely linked to RM158 [17]. However, the precise roles of these previously identified genes in disease resistance and susceptibility had not been well characterized. Therefore, there was a need for the identification of additional QTLs/genes that confer resistance to BLS in rice to more fully characterize disease mechanisms. Additionally, molecular marker-assisted selection (MAS) required an explicit understanding of the genetic architecture of agronomic traits [18], so more complete identification of QTLs was required for breeding applications.

In recent years, advances in genomic and transcriptomic technologies had provided powerful tools to investigate the molecular basis of plant-pathogen interactions. Microarray experiments, RNA sequencing (RNA-seq), and other high-throughput methods had enabled researchers to analyze the global gene expression patterns of both the host plant and the pathogen during infection, revealing key genes and pathways involved in the disease process. A microarray experiment was conducted to analyze changes in genome-wide gene expression in response to Xoc at the early stage of infection in both the rice transgenic line and its wild type [19]. One of the differentially expressed pathogenesis-related genes (DEPGs), *NRRB*, was found to play a role in rice-Xoc interactions [20]. A novel ankyrin-like protein, *AnkB*, was identified in Xoc and can invade host leaves via stomata and wounds. The type three secretion system (T3SS) of Xoc was found to be pivotal to its pathogenic lifestyle [21]. The effector protein *AvrRxo1*, an ATP-dependent protease, can enhance the virulence of Xoc and inhibit stomatal immunity in rice by targeting and degrading the rice *OsPDX1*, which was involved in pyridoxal phosphate synthesis, thereby reducing vitamin B6 levels in rice [22]. A rice multi-parent advanced generation inter-cross (MAGIC) population was used to map QTLs conferring resistance to BLS and another major rice disease, bacterial blight (BB) [23]. A genome-wide association study (GWAS) analysis was conducted on a collection of 236 diverse rice accessions, mainly *indica* varieties, allowing identification of 12 quantitative trait loci (QTLs) on chromosomes 1, 2, 3, 4, 5, 8, 9 and 11, that conferred resistance to five representative isolates of Thai Xoc [24]. GWAS mapping was also conducted to study BLS resistance in rice, and resources were identified with strong resistance and significant SNPs that can potentially be used for breeding BLS-resistant rice [25]. Despite these advances additional work was required to identify new QTLs/genes for BLS resistance in rice and confirm the elite alleles for their utilization in modern molecular breeding.

In this study, we conducted a GWAS to identify resistance to bacterial leaf streak (RBLS) using 747 cultivated rice accessions from the 3,000 Rice Genome (3 K-RG) project [2, 26]. We performed integrated gene annotation and genetic variation, homology, haplotype, and transcriptome analysis to identify candidate functional genes and possible causal polymorphisms for BLS in rice. Our results provided insights into the genetic architecture of BLS, and markers derived from the newly identified genes will be useful in improving resistance to BLS for modern molecular rice breeding.

## Results

### Population structure and phenotypic variation in RBLS traits among 747 cultivated rice accessions

**Fig. 1:**
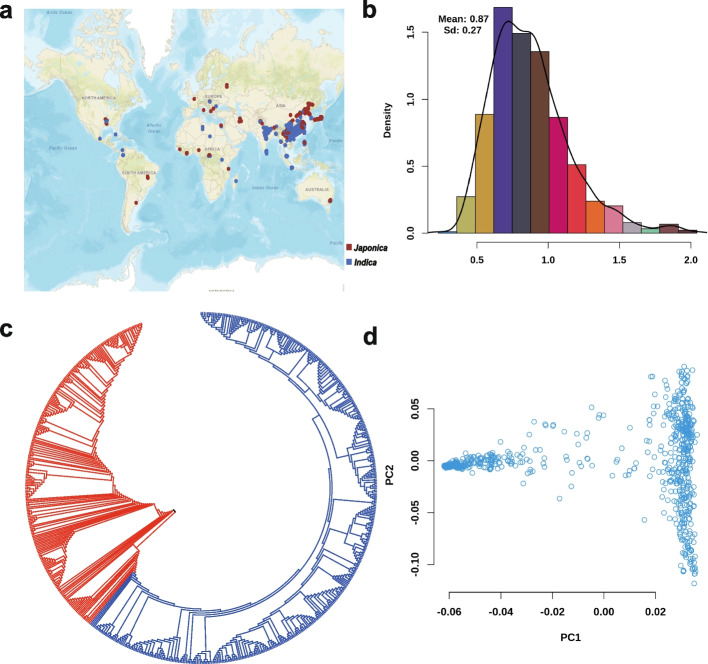
Population genomic analyses and phenotype statistics for RBLS in rice. **a**. The geographical distribution of 747 cultivated rice accessions using the R package “leaflet”, and the map was based on “Esri.WorldStreetMap” provided by OpenStreetMap. Dark red and light blue dots on the world map represented *japonica* and *indica*, respectively. **b**. Distribution of resistance to bacterial leaf streak (RBLS) in the full population. **c**. Phylogenetic tree of all accessions inferred from whole-genome SNPs. Major clades were indicated, and dark red and light blue lines represented *japonica* and *indica*, respectively. **d**. Principal component analysis (PCA) for different subpopulations

The 747 accessions were collected and a map was plotted using their latitude and longitude based on their clear geographic distribution (Fig. 1a, Additional file 2: Table S1). The Population structure and phenotypic variation in RBLS traits among 747 cultivated rice accessions were analyzed (Fig. 1b-d, Additional file 2: Table S1). We conducted principal component analysis (PCA) using 2.86 million SNPs to assess the population structure of 747 accessions (Fig. 1d). Our analysis revealed a distinct and profound subpopulation structure within this germplasm collection. The PCA indicated that the accessions were classified into two distinct subspecies groups: *Oryza sativa* ssp. *indica* (comprising 458 accessions) and *japonica* (comprising 289 accessions). The first two principal components (PCs) accounted for almost half of the genetic variation, with PC1 and PC2 accounting for 38% and 7% of the total genetic variation, respectively. Furthermore, the neighbor-joining tree also demonstrated a clear subpopulation structure within the 747 accessions (Fig. 1c).

We utilized ADMIXTURE software [27] to investigate the population structure and genotype structure of the 747 accessions. The analysis was based on the maximum likelihood estimation model and cross-validated for the number of subpopulations (K) to determine the optimal number of ancestral components (K = 1–10). Our results from the structural simulation analysis revealed that the cross-validation error (CV) was minimized when K = 2 (Additional file 1: Fig. S1). Therefore, we selected a k value of 2 to evaluate the genetic structure of the 747 rice genotypes. The distinct levels of K illustrated a clear separation between the *indica* and *japonica* subpopulations when K = 2 (Additional file 1: Fig. S1). As a result, we utilized the full population, as well as the *indica* and *japonica* subpopulations, to conduct further phenotypic analysis and GWAS.

The resistance to rice bacterial leaf streak displayed a variation that ranged from 0.416 to 2.042 in the full population and *indica* varieties, and from 0.421 to 1.816 in *japonica* varieties. Our phenotypic data exhibited a normal distribution and were deemed suitable for association analysis (Fig. 1b).

### GWAS and QTL identification of resistance to rice bacterial leaf streak

**Fig. 2:**
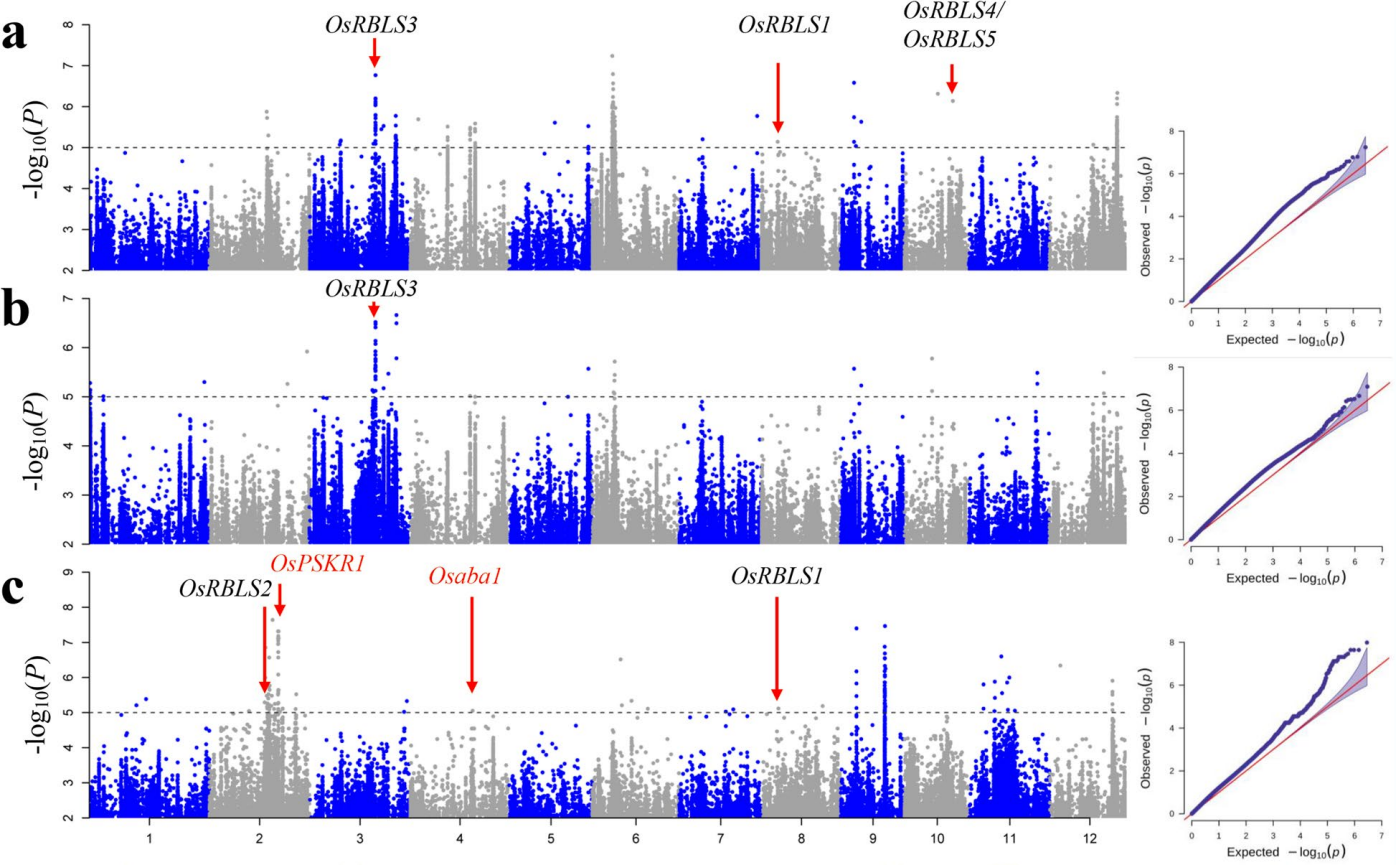
GWAS results of RBLS in different populations. Manhattan plots and Quantile–quantile plots for GWAS in full (**a**), *indica* (**b**), and *japonica* (**c**) using FarmCPU. The red and black genes indicated known genes and candidate genes involved in the RBLS and described in the text, respectively. A dotted horizontal line for each figure indicates the significance threshold (*P* = 10^−5^)

We conducted GWAS for the full population and the *indica* and *japonica* subpopulations, using the phenotypic data of resistance to rice bacterial leaf streak and sequence data from the 747 rice accessions. We employed the general linear model (GLM), mixed linear model (MLM), and fixed and random model circulating probability unification (FarmCPU) implemented in rMVP software [28]. To minimize false positives arising from population structure, we compared the quantile–quantile plots from the three models for BLS resistance for each population and determined that FarmCPU was more suitable than GLM and MLM. The first three principal components were used as covariates within the GWAS model to account for subpopulation structure. We established the suggestive significant threshold at -log (*P*) = 5 to detect significant association signals. For each GWAS result, we identified SNPs with *P*-values lower than the threshold (Fig. 2, Additional file 1: Figs. S2-S6).

Based on this criterion, we identified a total of 89 signals for RBLS across the full, *indica*, and *japonica* populations, with 28, 21, and 40 signals detected in the full, *indica*, and *japonica* populations, respectively (Fig. 2, Table 1, Additional file 3: Table S2). The QTLs detected by GWAS offered valuable insights into the genetic architecture of the observed phenotypic variations. To further investigate these associations for RBLS, we compared the QTLs identified by GWAS with previous linkage mapping results. Our analysis revealed that nine QTLs associated with RBLS had been previously identified in genetic linkage mapping studies [16, 29, 30].

**Table 1:** Significant association signals for RBLS in the full population detected by rMVP using FarmCPU model

Population	QTL Name	Chr	Lead SNP	-LOG(*P*)	PVE (%)	Previous QTL	Reference
Full population	*qRBLS2-1*	2	Chr2_20546456	5.88	7.13		
	*qRBLS2-2*	2	Chr2_21134213	5.30	10.83		
	*qRBLS3-1*	3	Chr3_10936852	5.07	11.31		
	*qRBLS3-2*	3	Chr3_11296448	5.17	6.41		
	*qRBLS3-3*	3	Chr3_23066648	5.09	8.11		
	*qRBLS3-4*	3	Chr3_23793189	6.77	9.47		
	*qRBLS3-5*	3	Chr3_25892532	5.45	8.60		
	*qRBLS3-6*	3	Chr3_26684482	5.53	7.79	*qnBLS3.2*	[29]
	*qRBLS3-7*	3	Chr3_31067057	5.77	6.34		
	*qRBLS3-8*	3	Chr3_31311287	5.18	8.17		
	*qRBLS4-1*	4	Chr4_2781244	5.69	8.13		
	*qRBLS4-2*	4	Chr4_13347526	5.51	12.12		
	*qRBLS4-3*	4	Chr4_21444248	5.48	6.74		
	*qRBLS4-4*	4	Chr4_23247915	5.59	6.84		
	*qRBLS5-1*	5	Chr5_16443814	5.61	11.57		
	*qRBLS5-2*	5	Chr5_28500512	5.52	10.95		
	*qRBLS6-1*	6	Chr6_7168224	7.24	13.73		
	*qRBLS6-2*	6	Chr6_8296955	5.97	15.34		
	*qRBLS7-1*	7	Chr7_8447328	5.20	10.07		
	*qRBLS7-2*	7	Chr7_28067617	5.77	12.75		
	*qRBLS8-1*	8	Chr8_5783713	5.14	8.09	*qnBLS8.4*	[29]
	*qRBLS9-1*	9	Chr9_4823765	6.58	12.49		
	*qRBLS9-2*	9	Chr9_5493858	5.04	8.32		
	*qRBLS9-3*	9	Chr9_7419876	5.63	9.78		
	*qRBLS10-1*	10	Chr10_12037931	6.31	6.46		
	*qRBLS10-2*	10	Chr10_17415410	6.14	8.33	*QTL1*	[30]
	*qRBLS12-1*	12	Chr12_15792613	5.07	9.68		
	*qRBLS12-2*	12	Chr12_24430488	6.34	7.25		

### Cloned genes resistance to rice bacterial leaf streak

To confirm our findings, we first checked whether the reported genes were located in the candidate QTLs. Subsequently, we extracted all genes that contained significant SNPs within each QTL and evaluated their annotations, homologous gene functions, linkage mapping in two bi-parental crosses, sequencing analysis, and distances from peak SNPs to identify the candidate genes in QTLs. Our GWAS identified two cloned genes related to RBLS located in QTLs, namely, *OsPSKR1* (Phytosulfokine receptor 1) [31] and *Osaba1* (Zeaxanthin epoxidase) [32] (Fig. 2, Additional file 3: Table S2).

**Fig. 3:**
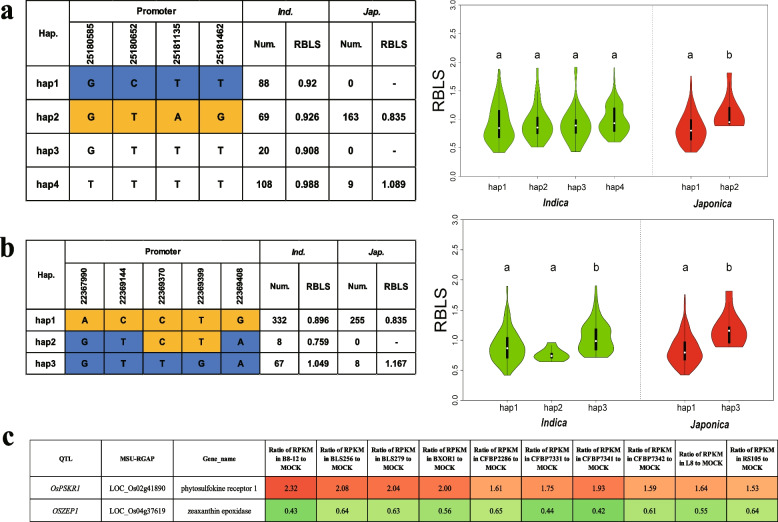
Exploration of two cloned genes for RBLS. **a** The cloned genes for RBLS and heat map of the ratio of RPKM. Different colors show ratio of RPKM in the *Oryza* *sativa* L. ssp. *japonica* cv. Nipponbare leaves at 48 h after inoculation with 10 geographically diverse strains of *Xanthomonas* *oryzae* pv. *oryzicola* and mock inoculated. Rows of the heat map correspond to the two cloned genes for RBLS listed on the left of the table. Haplotype analysis of *OsPSKR1* (**b**) and *Osaba1* (**c**). Different haplotypes and a comparison of RBLS among haplotypes of *OsPSKR1* and *Osaba1* in the *indica* and *japonica* subgroups. The green violins represent *indica*, the red violins represent *japonica* rice, and different letters indicate significant differences (*P* < 0.05) detected by one-way ANOVA

*OsPSKR1* had been shown to rescue root growth and influence susceptibility to *Pseudomonas syringae* pv. *DC3000* in Arabidopsis *pskr1-3* mutants. Moreover, the expression of *OsPSKR1* was found to be up-regulated following inoculation with RS105, a strain of Xoc that causes bacterial leaf streak in rice [31]. Based on our sequence data, we found four SNPs (*P* ≤ 10^–2^) located in the promoter region of *OsPSKR1* (Fig. 3a). We conducted haplotype analysis for *OsPSKR1* using the genotypes of these four SNPs for the 747 accessions, and we identified four haplotypes (hap1, hap2, hap3, and hap4) (Fig. 3a). In the *japonica* subpopulation, we observed a significant difference in the mean value of RBLS between hap2 and hap4; the mean RBLS for hap4 (1.089, 9 accessions) was higher than that for hap2 (0.835, 163 accessions). However, no significant difference was found between the four haplotypes in the *indica* subpopulation. Therefore, we concluded that *OsPSKR1* was a functional gene that regulates RBLS, and hap2 of *OsPSKR1* was identified as the superior genotype. Increasing the frequency of hap2 in the *japonica* subpopulation could enhance resistance to rice bacterial leaf streak. In summary, hap2 of *OsPSKR1* could be utilized to improve resistance to RBLS in rice (Fig. 3a).

The *Osaba1* mutant has been shown to be resistant to Xoc [32]. We identified five significant SNPs located in the promoter region of *Osaba1*. Haplotype analysis was conducted for *Osaba1* using genotypic data from the 747 accessions, which revealed a total of three haplotypes (hap1, hap2, and hap3) in both the *indica* and *japonica* subpopulations (Fig. 3b). In the *indica* subpopulation, hap1 and hap2 of *Osaba1* exhibited a significant difference in RBLS compared to hap3, with the mean RBLS of hap3 (1.049, 67 accessions) being higher than that for hap1 (0.896, 332 accessions) and hap2 (0.759, 8 accessions). Therefore, we tentatively identified this gene as a functional gene that controls bacterial leaf streak in rice, with hap1 and hap2 of *Osaba1* being identified as the superior genotypes. However, we also observed a higher proportion of superior haplotypes for this gene in both *indica* and *japonica* rice, indicating that these superior haplotypes have already been widely utilized in modern breeding processes (Fig. 3b).

### Transcriptome analysis of candidate genes

**Fig. 4:**
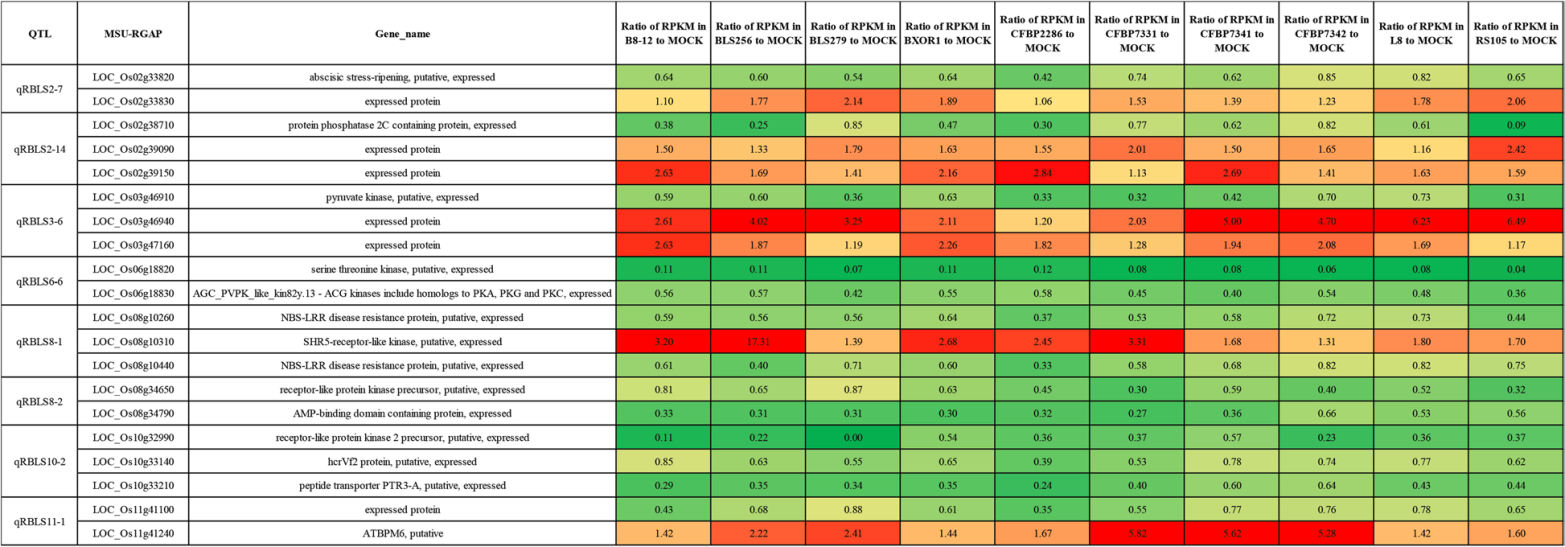
Candidate genes for RBLS and heat map of the ratio of RPKM. Different colors show the ratio of RPKM in leaves of Nipponbare 48 h after inoculation with 10 geographically diverse strains of Xoc and mock-inoculated. The rows of the heat map correspond to the 20 candidate genes for RBLS listed on the left of the table

To ensure the reliability of our results, we conducted transcriptome analysis on Nipponbare leaves 48 h after inoculation with 10 geographically diverse strains of Xoc. We utilized three biological replicates for each condition, and three replicates of mock-inoculated *O. sativa* were used as controls. We compared the reads per kilobase per million mapped reads between the samples inoculated with the Xoc strains and the mock-inoculated samples, denoted as RPKM_Xoc_ and RPKM_Mock_, respectively. The genes detected in the GWAS for RBLS were classified as up-regulated or down-regulated genes based on their RPKM_Xoc_/RPKM_Mock_ values. Genes with values greater than 1.5 were classified as up-regulated, while those with values lower than 0.67 were classified as down-regulated, between the inoculation with 10 geographically diverse strains of Xoc and the mock-inoculated samples (Figs. 3c and 4). As previously described, our GWAS results identified two cloned genes that may be functional genes for RBLS in the *japonica* population. *OsPSKR1* was identified as an up-regulated gene, with the ratios of its RPKM values between the 10 geographically diverse strains of Xoc and the mock-inoculated samples being greater than 1.5. The ratio of RPKM in B8-12 to the mock sample for *OsPSKR1* was 2.32, and the lowest ratio of RPKM in RS105 to mock for *OsPSKR1* was 1.53. *Osaba1* was identified as a down-regulated gene, with the ratios of its RPKM values between the 10 geographically diverse strains of Xoc and the mock-inoculated samples being all lower than 0.67. The lowest ratio for *Osaba1* was 0.42, which was the ratio of RPKM in CFBP7341 to the mock sample. The largest ratio of RPKM in CFBP2286 to mock for *Osaba1* was 0.65 (Fig. 3c). These results were in agreement with previous reports, indicating that the transcriptomic analyses were trustworthy and could be employed to further identify potential candidate genes for the QTLs detected by GWAS.

With the reliability of the transcriptomic analyses established, we proceeded to identify potential candidate genes within the QTL for RBLS by comparing the RPKM values of genes within each QTL detected by GWAS between the samples inoculated with 10 geographically diverse strains of Xoc and the mock-inoculated samples. We utilized the transcriptomic data to identify 20 candidate genes that corresponded to the eight QTLs detected by GWAS. Among these, seven genes were up-regulated, while 13 genes were found to be down-regulated (Fig. 4). Based on the transcriptomic analysis, these genes were the most likely candidate genes for RBLS among the 20 candidates we identified.

To investigate the function of these candidate genes, the same approach of KEGG pathway enrichment analysis for cloned and candidate genes were applied. The cloned and candidate genes were enriched for KEGG pathway (Additional file 1: Figs. S7-S8). The terms “MAPK signaling pathway—plant”, “Plant hormone signal transduction” and “Biosynthesis of secondary metabolites” from cloned genes (Additional file 1: Fig. S7), and “MAPK signaling pathway—plant”, “Biosynthesis of secondary metabolites” and “Metabolic pathways” from candidate genes (Additional file 1: Fig. S8) were both related to development and resistance. Taken together, these candidate genes were most likely the genes that control RBLS, and could provide theoretical guidance for subsequent research on disease resistance in rice.

### Candidate gene analysis in QTLs

**Fig. 5:**
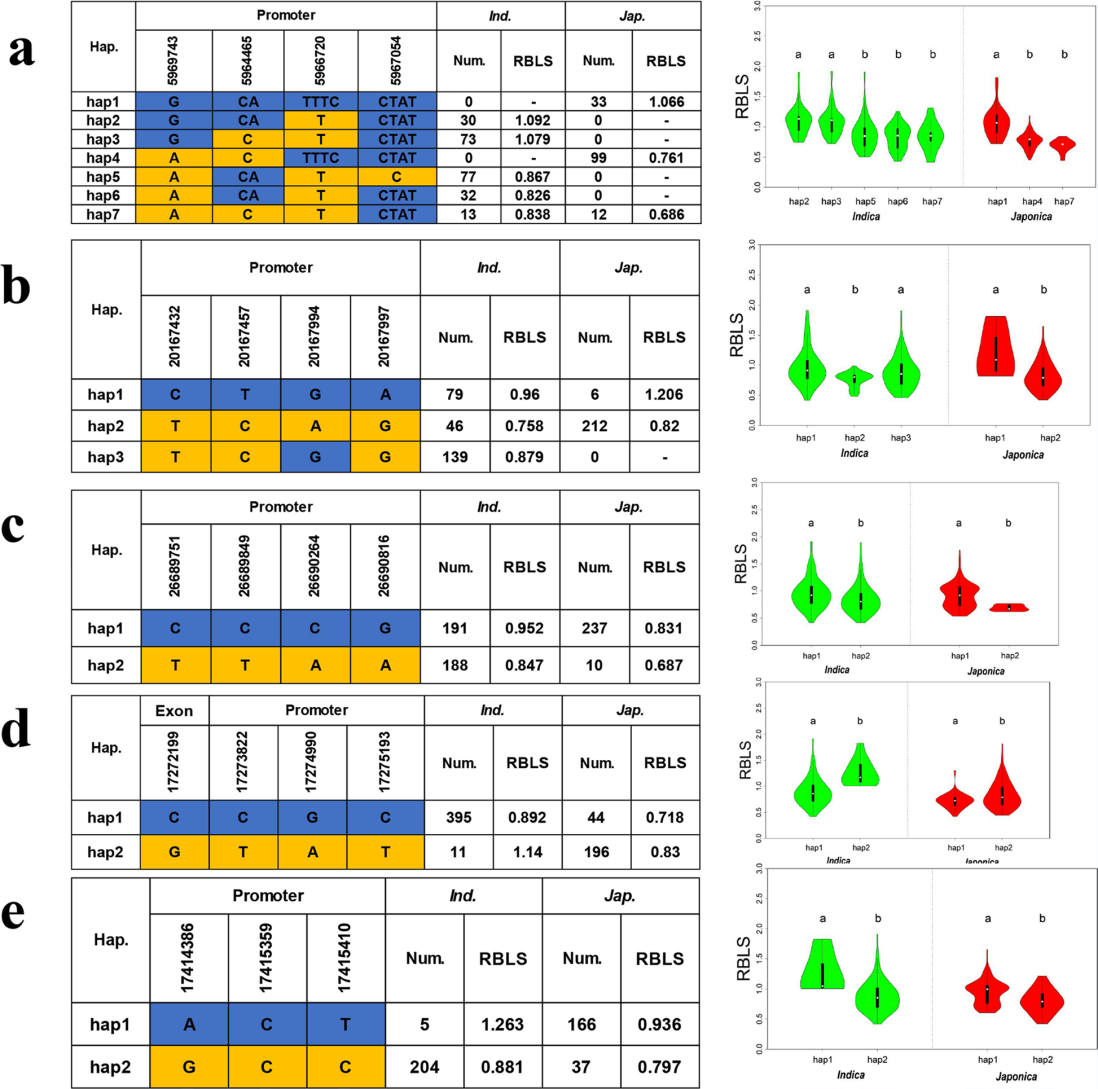
Investigation of five candidate genes for RBLS. Different haplotypes and a comparison of RBLS between the haplotypes of *OsRBLS1* (**a**), *OsRBLS2* (**b**), *OsRBLS3* (**c**), *OsRBLS4* (**d**) and *OsRBLS5* (**e**) in the *indica* and *japonica* subgroups. The green violins represent *indica*, the red violins represent *japonica* rice, and different letters indicate significant differences (*P* < 0.05) detected by one-way ANOVA

To gain a deeper understanding of the potential candidate genes, we conducted haplotype analysis for these genes. Our analysis revealed significant differences in the mean RBLS values between haplotypes for five of the genes (Fig. 5).

LOC_Os08g10260 was located in qRBLS8-1 and encoded a NBS-LRR disease resistance protein. We found four SNPs (*P* ≤ 10^–2^) within the promoter region of the gene. Haplotype analysis was conducted for LOC_Os08g10260 using the genotypes of these four SNPs for all 747 accessions, ultimately leading to the identification of seven haplotypes (Fig. 5a). In the *indica* subpopulation, we observed a significant difference in RBLS between hap2 (1.092, 30 accessions) and hap3 (1.079, 73 accessions) compared to hap5 (0.867, 77 accessions), hap6 (0.826, 32 accessions), and hap7 (0.838, 13 accessions). Hap2 and hap3 exhibited higher mean RBLS values than hap5, hap6, and hap7. In *japonica*, we observed a significant difference in RBLS between hap1 (1.066, 33 accessions), and hap4 (0.761, 99 accessions), hap7 (0.686, 12 accessions), with the mean RBLS value for hap1 greater than that of hap4 and hap7. Based on our findings, we concluded that LOC_Os08g10260, which we named *OsRBLS1*, is a functional gene that controls RBLS. Hap4, hap5, hap6, and hap7 of *OsRBLS1* were identified as the superior genotypes. Thus, increasing their frequency in both the *indica* and *japonica* subpopulations could enhance resistance to rice bacterial leaf streak (Fig. 5a).

*ASR1* (LOC_Os02g33820), located in *qRBLS2-7*, encoded an abscisic stress-ripening gene. We found four SNPs (*P* ≤ 10^–2^) within the promoter region of *ASR1*. Prior research had demonstrated that overexpression of *ASR1* in transgenic rice plants led to increased tolerance to both drought and cold stress. Furthermore, *ASR1* and *ASR5* had been shown to function complementarily in the regulation of gene expression in response to aluminium (Al) toxicity [33, 34]. We conducted haplotype analysis for *ASR1* using the genotypes of these four SNPs for all 747 accessions, ultimately leading to the identification of three haplotypes (Fig. 5b). Within the *indica* subspecies, we noted a significant difference in RBLS between hap1, hap3, and hap2. The mean RBLS value of hap2 (0.758, 46 accessions) was lower than those for hap1 (0.96, 79 accessions) and hap3 (0.879, 139 accessions). In the japonica subspecies, a significant difference in RBLS was observed between hap1 and hap2, with hap1 exhibiting a higher mean RBLS value than hap2. Based on our results, we concluded that *ASR1* (LOC_Os02g33820), which we named *OsRBLS2*, was a functional gene that controls RBLS. Regarding *OsRBLS2*, hap2 was identified as the superior genotype. Thus, increasing the frequency of hap2 in the *indica* subpopulation could enhance resistance to rice bacterial leaf streak (Fig. 5b).

We observed a significant difference in RBLS between hap1 and hap2 of LOC_Os03g47160, LOC_Os10g32990, and LOC_Os10g33210 within both the *indica* and *japonica* subpopulations. LOC_Os03g47160 was located within qRBLS3-1 and encoded an expressed protein that we named *OsRBLS3*. LOC_Os10g32990 and LOC_Os10g33210 were both located in *qRBLS10-2*. LOC_Os10g32990 encoded a precursor of receptor-like protein kinase 2, named *OsRBLS4*. LOC_Os10g33210, on the other hand, encoded a peptide transporter PTR3-A that we named *OsRBLS5*. In both the *indica* and *japonica* subpopulations, the mean RBLS values of hap2 for *OsRBLS3* and *OsRBLS5* were significantly lower than those for hap1. While, the mean RBLS value of hap2 of *OsRBLS4* was significantly higher than that for hap1 in both *indica* and *japonica*. Therefore, we concluded that *OsRBLS3*, *OsRBLS4*, and *OsRBLS5* were functional genes that control RBLS. The hap2 of *OsRBLS3* and *OsRBLS5*, and hap1 of *OsRBLS4* were superior genotypes. Increasing the frequency of these genotypes in either the *indica* or *japonica* subpopulations could improve resistance to rice bacterial leaf streak (Fig. 5c-e). By employing GWAS and linkage mapping in conjunction with sequence analysis, haplotype analysis, and transcriptome analysis, we identified 20 QTLs and five candidate genes that control resistance to bacterial leaf streak in rice. We also determined the superior haplotypes of these genes and assessed their breeding potential in both the *indica* and *japonica* subspecies. These genes could be useful in further investigations aimed at elucidating the genetic mechanisms underlying resistance to bacterial leaf streak in rice.

## Discussion

### Genetic factors contributing to resistance to bacterial leaf streak in rice

Efforts to develop BLS-resistant rice varieties necessitated the exploration and utilization of genes associated with RBLS. To date, several genes related to bacterial leaf streak disease in rice have been cloned [31, 32, 35–38]. The CRISPR/Cas9 system was used to edit the promoter region of the BLS susceptibility gene *OsSULRT3;6*, which effectively enhanced the resistance of rice to BLS [35]. The transcription activator-like effector *Tal2b* could bind directly to the promoter region of *OsF3H03g*, which encoded a 2-oxoglutarate-dependent dioxygenase in rice. *OsF3H03g* can negatively regulated salicylic acid (SA)-related defense by directly reducing SA, and promotes susceptibility to both Xoc and Xoo in rice [36]. Compared with the wild-type, overexpression (OE) lines of *OsHsp18.0-CI* showed enhanced resistance to RS105, whereas repression lines showed compromised resistance to RS105 [37]. Compared to wild-type controls, transgenic rice plants overexpressing *DEPG1* exhibited enhanced susceptibility to Xoc [39]. Two cloned genes related to RBLS, *OsPSKR1* and *Osaba1*, were located in the QTLs detected in our GWAS.

### Environmental factor affecting disease incidence

BLS tended to thrive in hot and humid conditions, particularly during periods of frequent rainfall, and can cause significant yield losses in rice. In years with frequent storms, these losses can average up to 50% [8, 9, 40]. Physiological differences among pathogenic races may contribute to the variation in virulence of bacterial leaf streak pathogens [41]. The genetic mechanisms of genes for RBLS might vary depending on the plant material, bacterial strain, or identification method used. There has been significant variation in the mapping of genes related to bacterial leaf streak resistance. For example, resistance in material BJI to the tested pathogen was conferred by three pairs of recessive genes, as determined by spray inoculation during the seedling stage [42]. The resistance of hybrid rice to the tested pathogen was dominant, but the susceptibility was recessive, as determined by spray inoculation during the seedling stage, indicating that the resistance of the restorer lines determined the resistance of the hybrid material [43]. The resistance of Duar and IR36 to the tested pathogen was identified by needle inoculation, and was suggested to be controlled by two pairs of recessive genes [44]. Eight materials were randomly selected from 57 resistant wild rice materials for hybridization and backcrossing with 9311 and resistance was found to be inherited recessively [45]. BC_2_ generations were constructed using H359 and Jiannong 8, and resistance to bacterial leaf streak disease was found to be controlled by multiple genes as a quantitative trait [11]. Similarly, another study found that inheritance of resistance to bacterial leaf streak disease was controlled by multiple genes as a quantitative trait [12]. Four resistant rice varieties, Dular, IR1545-339, IR26, and IR36, as well as a susceptible variety, Jingang 30, were used for hybridization and backcrossing and the resistance gene in IR1545-339 was found to be non-allelic [46]. A highly pathogenic bacterial strain, S-103, was used to inoculate resistant 90IRBBN44, and genetic analysis revealed that the resistance in 90IRBBN44 was controlled by a pair of recessive genes [41]. An F_2_ population was constructed using the moderately resistant rice variety Minghui 86 and the highly resistant rice variety Jiafu as parents and a resistance quantitative trait locus (QTL) was detected between the second chromosome markers RM279-RM154 [47]. A mapping population was constructed using the resistant rice material Acc8558 and the susceptible rice material H359 through selfing and backcrossing. The resistance quantitative trait locus (QTL) *qBlsr5a* was shown to be located on the fifth chromosome [14]. The BLS resistance quantitative trait locus (QTL) *qBlsr3d* in rice was verified to be controlled by quantitative inheritance by constructing a H359-BLSR3D single-chromosome segment substitution line [48]. The resistance gene *bls2* was inherited in a recessive manner and is preliminarily mapped between chromosome 2 markers SL03 and SL04, with a range of approximately 4 cM based on the wild rice resistant DY19 and SSR molecular markers [16]. The BLS resistance gene in the international rice variety BJ1 was located using the BSA method. Resistance was found to be controlled by a pair of recessive genes located on the chromosome 10 at a distance of approximately 48.8 cM and tightly linked to marker RM158 [17]. A doubled haploid (DH) population was constructed using the parents Taichung Native 1 and Chunjiang 06 and four effective quantitative trait loci (QTL) were identified through needle inoculation during the seedling stage on the 2, 4, 5, and 8 chromosomes of rice [49].

Resequencing-based genotyping revealed a wide range of natural variations, enabling the exploration of functional genes and elite alleles (superior haplotypes) in natural populations through GWAS [50–52]. In our QTL analysis using FarmCPU, we detected previously identified RBLS genes *OsPSKR1* and *Osaba1*. However, many genes previously identified using mutants and gene differential expression as related to BLS resistance [31, 32, 35–38] did not exhibit mutations or variations in our populations. As a result, it may be necessary to investigate additional populations to identify distinct functional genes.

### Implications for sustainable rice production

While naturally immune rice varieties for BLS had yet to be discovered, identifying bacterial leaf streak resistance genes provided an opportunity to develop highly resistant varieties capable of reducing losses in rice yield and quality caused by the disease. Consequently, identifying resistance genes can facilitate the breeding of plants for the scientific, effective, and rational control of BLS disease. Although many BLS resistance genes were previously identified using mutants and gene differential expression [31, 32, 35–38], these genes exhibited no or few variations in natural populations with few resistance genes that could be effectively applied in breeding practice. In this study, we identified two cloned genes related to RBLS, *OsPSKR1* and *Osaba1*, located in the QTLs detected in our GWAS. We identified 20 QTLs and five candidate genes associated with resistance to BLS in rice through a combination of linkage mapping, sequence analysis, haplotype analysis, and transcriptome analysis. Moreover, we determined the superior haplotype and breeding potential of these genes in both *indica* and *japonica* rice varieties. These genes could be employed in future investigations aimed at elucidating the genetic mechanisms underlying resistance to bacterial leaf streak in rice.

## Conclusion

To summarize, we analyzed the population structure of 747 rice accessions and evaluated their phenotypes 20 days after inoculation with Xoc strain GX01. We then conducted GWAS on the full population and the *indica* and *japonica* subpopulations based on both the phenotypic resistance to rice bacterial leaf streak and the sequence data of the 747 rice accessions. Our analysis led to the identification of 20 QTLs associated with RBLS in rice. Subsequently, we used a combination of linkage mapping, sequence analysis, haplotype analysis, and transcriptome analysis to identify five candidate genes that control resistance to BLS in rice. We also determined their superior haplotypes and breeding potential in both *indica* and *japonica* rice varieties. These findings suggested that these genes could be employed in future studies aimed at elucidating the genetic mechanisms underlying resistance to bacterial leaf streak in rice.

## Methods

### Materials and sequencing data

The rice diversity panel comprised 747 cultivated rice accessions sourced from the 3 K Rice Genome (3 K-RG) [2, 26]. This panel included 290 genotypes from the mini-core collection, which was initially selected from a core set of 4,310 accessions [53], and 457 lines from the International Rice Molecular Breeding Network [54]. The two collections consisted of accessions from 45 countries, encompassing major rice-growing regions worldwide (Table S1). A map for 747 accessions was plotted using the R package “leaflet” (https://rstudio.github.io/leaflet/), and the map was based on “Esri.WorldStreetMap” provided by OpenStreetMap, with latitude and longitude of based on their clear geographic distribution. Transcriptomic data were obtained from the NCBI (https://www.ncbi.nlm.nih.gov/gds) with series accession ID: PRJNA280380 [55].

### Phenotypic data for RBLS

A total of 747 varieties were grown under field conditions in Nanning, Guangxi, southern China, with a plant spacing of 20 cm x 30 cm, and each variety was replicated twice. The bacterial strains of rice bacterial leaf streak were obtained from GX01, which was isolated at the Guangxi Academy of Agricultural Sciences. At the maximum tillering stage, the 747 varieties were inoculated with Xoc using the acupuncture method [10]. Phenotypic assessments were conducted 20 days after inoculation (DAI), and the diseased leaf areas were measured using the Standard Evaluation System for Rice (5th Edition, 2014).

### Phylogenetic and population structure analysis

The genetic variation data for the 747 accessions, in the form of single nucleotide polymorphisms (SNPs), were obtained from the publicly available 3 K-RG database. The database comprised approximately 17 million highly credible SNPs and 2.4 million indels, which were aligned to the Nipponbare IRGSP 1.0 reference genome [2, 26].

After removing SNPs with missing rates > 30% and minor allele frequencies < 5% in the full, *indica*, and *japonica* populations, we identified 2,860,820 SNPs as a credible SNP set. To account for population structure, we performed principal component (PC) and kinship matrix analyses for the 747 accessions. To infer the basal group of rice, we used a total of 2,860,820 SNPs with a missing rate of ≤ 50% to construct a phylogenetic tree using the unweighted neighbor-joining (NJ) method with the 'phangorn' R package. For constructing the PC matrix, we used the first three principal components (PCs). To conduct PCA, we used R (version 4.0.3) and plotted the first three eigenvectors. We further called independent SNPs using Plink v1.90b4 with the parameter '–indep-pairwise 50 5 0.3'. This resulted in the effective numbers of independent SNPs being 113,223, 95,164, and 45,752 for the full, *indica*, and *japonica* populations, respectively [56]. To analyze the population structure of the rice accessions, we used the 113,223 independent SNPs to perform a maximum likelihood clustering analysis with ADMIXTURE (version 1.3) [27]. To estimate the genetic ancestry of each sample, we ran the cross-validation error (CV) procedure with varying levels of K (K = 1–10). Based on the cross-validation error, we determined that a value of K = 2 was optimal. We plotted the ADMIXTURE result using an R script.

### Genome-wide association study

We obtained a total of 2,860,820 SNPs with a missing rate of ≤ 50% and minor allele frequencies of ≥ 5%. To account for population structure, we performed principal component (PC) and kinship matrix analyses, using the first three PCs to construct the PC matrix. We performed a genome-wide association study (GWAS) to identify genetic variants associated with resistance to rice bacterial leaf streak (BLS) in the 747 accessions. And we used three different models: general linear model (GLM), mixed linear model (MLM), and Fixed and random model Circulating Probability Unification (FarmCPU). The GWAS was conducted using the SNP set and default settings in the rMVP software [28]. The strong linkage disequilibrium (LD) among SNPs resulted in non-independence, which caused the thresholds derived from the total number of markers to be too stringent for detecting significant associations. To address this issue, we calculated suggestive thresholds using the formula "-log10(1 / effective number of independent SNPs)", as previously described [50, 52, 57, 58]. And we determined the effective numbers of independent SNPs using PLINK [56], with a window size of 50, step size of 50, and r^2^ ≥ 0.3. The effective numbers of independent SNPs were found to be 113,223, 95,164, and 45,752 in the full population, and the *indica* and *japonica* subpopulations, respectively. To identify significant associations, we set the threshold at -log (*P*) = 5. We used genome structure annotation information from MSU-RGAP 7.0 to annotate nonsynonymous SNPs using SnpEff [59]. An in-house Perl script was used to separate these SNPs from all the identified SNPs in the 747 accessions.

### Haplotype analysis

For haplotype analysis, we used SNPs with a significance level of *P* ≤ 10^–2^ in the 2 k promoter and exons of the gene. Haplotypes that contained at least five varieties were used for statistical testing. We calculated the differences in phenotypic value between haplotypes of each gene using one-way ANOVA or Student's t-tests with an R script [1, 51, 52]. Duncan's multiple range tests were used for comparisons, when the results of one-way ANOVA were significant (*P* < 0.01).

### Transcriptome analysis

To identify candidate genes within the QTL associated with resistance to rice bacterial leaf streak, we performed transcriptome analysis on leaves of Nipponbare at 48 h after inoculation with 10 geographically diverse strains of Xoc, the causal agent of bacterial leaf streak. We used three biological replicates for each, and three replicates of mock-inoculated *O. sativa* as a control (PRJNA280380) [55]. We performed mapping of RNAseq reads and quantification of transcript abundance RPKM using Cufflinks. And we determined the thresholds of RPKM (reads per kilobase per million mapped reads) RPKM_Xoc_/RPKM_mock_ between inoculation with 10 geographically diverse strains of Xoc and mock inoculation, as well as the ratio of RPKM > 1.5 or < 0.67 in our transcriptomic analysis [52, 60]. Genes with RPKM_Xoc_/RPKM_mock_ > 1.5 were defined as up-regulated genes, and those with RPKM_Xoc_/RPKM_mock_ < 0.67 were defined as down-regulated genes.

### Supplementary Information

**Additional file 1:** **Fig. S1.** Population structure analyses of 747 rice accessions. **Fig. S2.** Quantile–quantile plots for the GWAS in full population using GLM, MLM and FarmCPU. **Fig. S3.** Quantile–quantile plots for the GWAS in *indica* population using GLM, MLM and FarmCPU. **Fig. S4.** Quantile–quantile plots for the GWAS in *japonica* population using GLM, MLM and FarmCPU. **Fig. S5.** GWAS results of RBLS in different populations using MLM. **Fig. S6.** GWAS results of RBLS in different populations using GLM. **Fig. S7.** Pathway enrichment of the cloned genes for RBLS. **Fig. S8. **Pathway enrichment of the candidate genes for RBLS.

**Additional file 2:** **Table S1.** Description of 747 rice accessions.

**Additional file 3:** **Table S2.** Significant association signals for RBLS in the *indica* and *japonica* populations detected by rMVP using FarmCPU model.

## Data Availability

The genomic data for 747 rice accessions from the 3 K-RG can be downloaded from http://snp-seek.irri.org. The sequencing data used in our analyses can be accessed through project accession PRJEB6180 on NCBI (https://www.ncbi.nlm.nih.gov/sra/?term=PRJEB6180). The public transcriptome data includes data for Nipponbare leaves at 48 h after inoculation with 10 geographically diverse strains of Xoc, with three biological replicates for each compared to three replicates of mock-inoculated *O. sativa* as the control (PRJNA280380). All data generated or analyzed during this study are included in this published article or its supplementary information files or are available from the corresponding authors on reasonable request.

## Abbreviations

BLS: Bacterial leaf streak
Xoc: *Xanthomonas* *oryzae* Pv. *oryzicola*
T3SS: Type three secretion system
VB6: Vitamin B6
GWAS: Genome-wide association study
QTL: Quantitative trait loci
MAS: Marker-assisted selection
3 K-RG: 3,000 Rice Genome
RBLS: Resistance to bacterial leaf streak
PCA: Principle component analysis
CV: Cross-validation error
GLM: General linear model
MLM: Mixed linear model
FarmCPU: Fixed and random model Circulating Probability Unification
SA: Salicylic acid
RPKM: Reads Per Kilobase per Million mapped reads
RPKM_Xoc_: RPKM of inoculation with 10 geographically diverse strains of *Xanthomonas* *oryzae* pv. *oryzicola*
RPKM_mock_: RPKM of inoculation with mock
