# Supplementary material for: Genetic-based dissection of resistance to bacterial leaf streak in rice by GWAS
Source: BMC Plant Biol. 2023 Aug 18;23:396. doi: 10.1186/s12870-023-04412-7 (PMC10436437; doi:10.1186/s12870-023-04412-7)
Supplement: Supplementary file 1 — Additional file 1: Fig. S1. Population structure analyses of 747 rice accessions. Fig. S2. Quantile–quantile plots for the GWAS in full population using GLM, MLM and FarmCPU. Fig. S3. Quantile–quantile plots for the GWAS in indica population using GLM, MLM and FarmCPU. Fig. S4. Quantile–quantile plots for the GWAS in japonica population using GLM, MLM and FarmCPU. Fig. S5. GWAS results of RBLS in different populations using MLM. Fig. S6. GWAS results of RBLS in different populations using GLM. Fig. S7. Pathway enrichment of the cloned genes for RBLS. Fig. S8. Pathway enrichment of the candidate genes for RBLS. [file 12870_2023_4412_MOESM1_ESM.docx]

**Supplementary Information**

**Title:** Genetic-based dissection of resistance to bacterial leaf streak in rice by GWAS

Xiaoyang Zhu^3†^, Lei Chen^1,4†^, Zhanying Zhang^3^, Jinjie Li^3^, Hongliang Zhang^3^, Zichao Li^3^, Yinghua Pan^1,4*^ & Xueqiang Wang^1,2*^

^1^ Hainan Yazhou Bay Seed Laboratory, Sanya, Hainan, 572025, PR China.

^2^ Zhejiang Provincial Key Laboratory of Crop Genetic Resources, College of Agriculture and Biotechnology, Zhejiang University, Hangzhou, 310058, China.

^3^ State Key Laboratory of Agrobiotechnology / Beijing Key Laboratory of Crop Genetic Improvement, China Agricultural University, Beijing, 100193, China.

^4^ Guangxi Key Laboratory of Rice Genetics and Breeding, Rice Research Institute, Guangxi Academy of Agricultural Sciences, Nanning 530007, China.

^†^ These authors have contributed equally to this work.

^*^ Correspondence should be addressed to Xueqiang Wang (Email: [wangxueqiang02@163.com](mailto:wangxueqiang02@163.com)) and Yinghua Pan (Email: panyinghua2008@163.com).

**Additional file 1. Fig. S1** Population structure analyses of 747 rice accessions.

**Additional file 1. Fig. S2** Quantile–quantile plots for the GWAS in full population using GLM, MLM and FarmCPU.

**Additional file 1. Fig. S3** Quantile–quantile plots for the GWAS in *indica* population using GLM, MLM and FarmCPU.

**Additional file 1. Fig. S4** Quantile–quantile plots for the GWAS in *japonica* population using GLM, MLM and FarmCPU.

**Additional file 1. Fig. S5** GWAS results of RBLS in different populations using MLM.

**Additional file 1. Fig. S6** GWAS results of RBLS in different populations using GLM.

**Additional file 1. Fig. S7** Pathway enrichment of the cloned genes for RBLS.

**Additional file 1. Fig. S8** Pathway enrichment of the candidate genes for RBLS.

**Additional file 2. Table S1.** Description of 747 rice accessions.

**Additional file 3. Table S2.** Significant association signals for RBLS in the *indica* and *japonica* populations detected by rMVP using FarmCPU model.


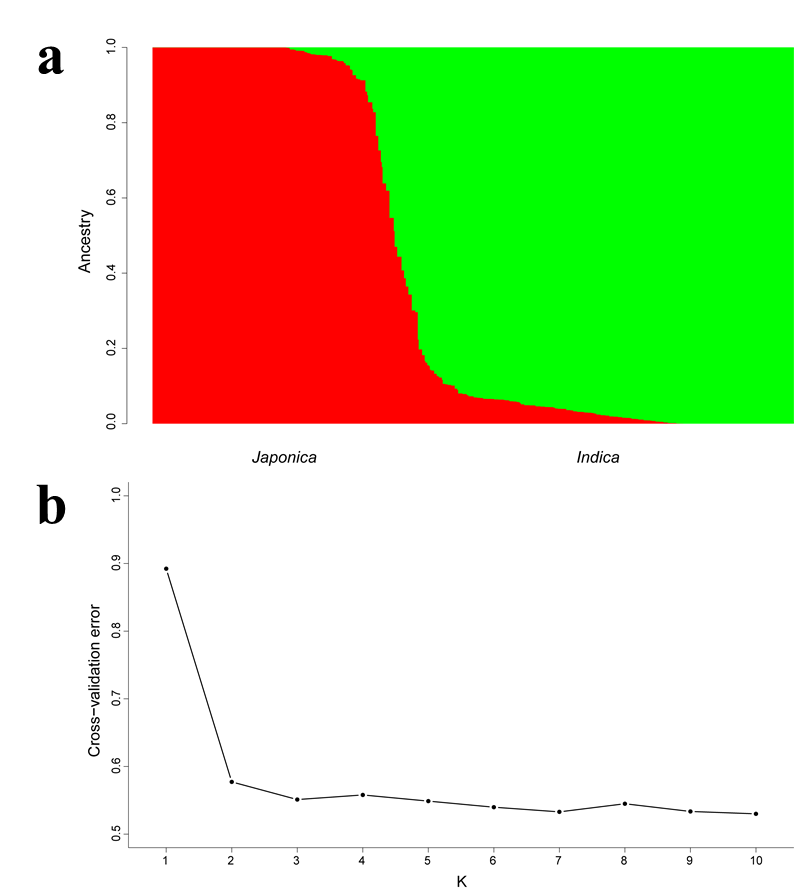


**Fig. S1** Population structure analyses of 747 rice accessions.


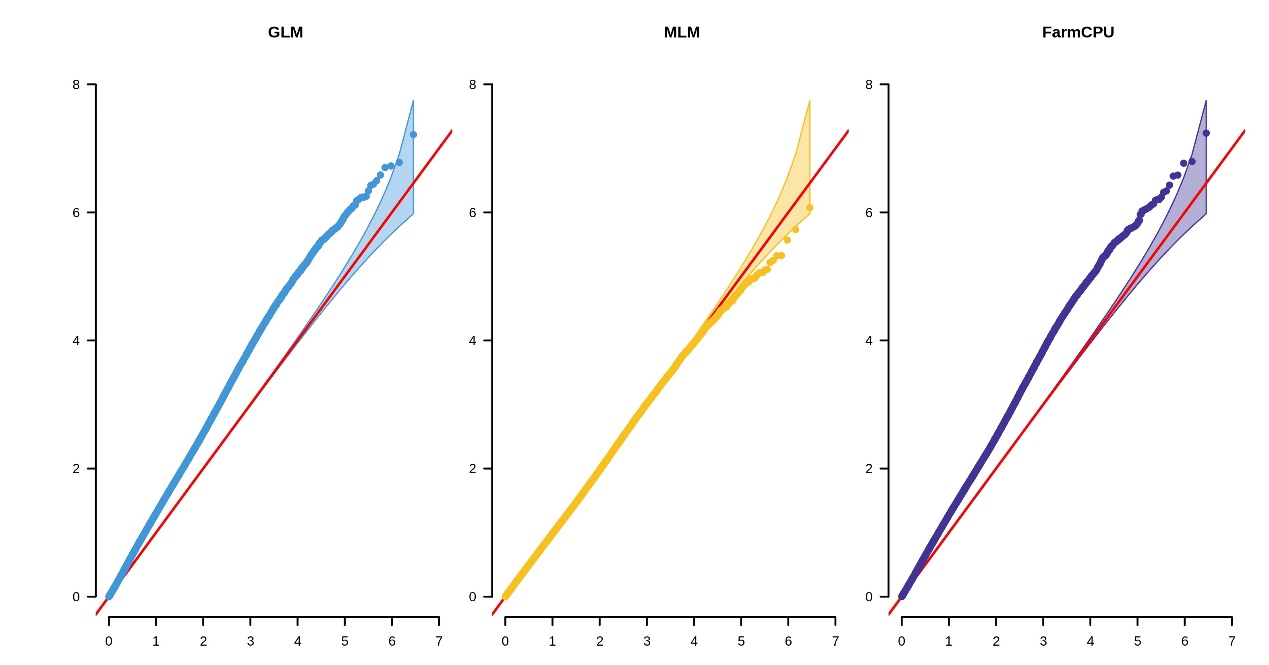


**Fig. S2** Quantile-quantile plots for the GWAS in full population using GLM, MLM and FarmCPU.


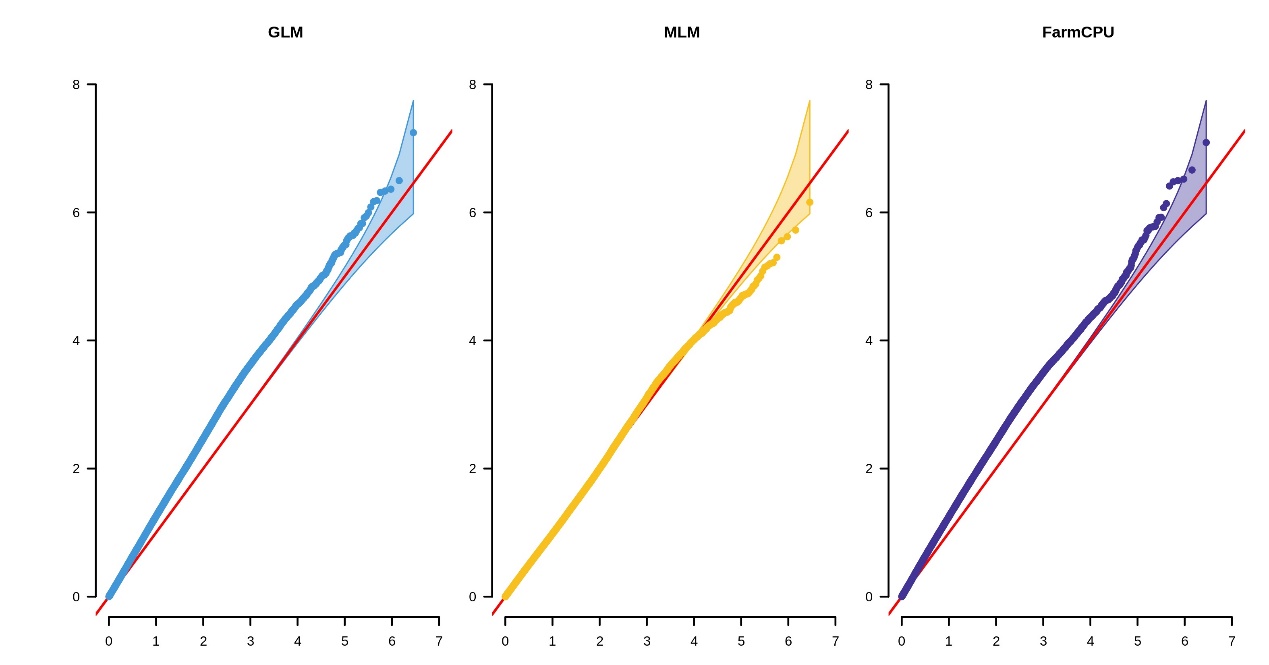


**Fig. S3** Quantile-quantile plots for the GWAS in *indica* population using GLM, MLM and FarmCPU.


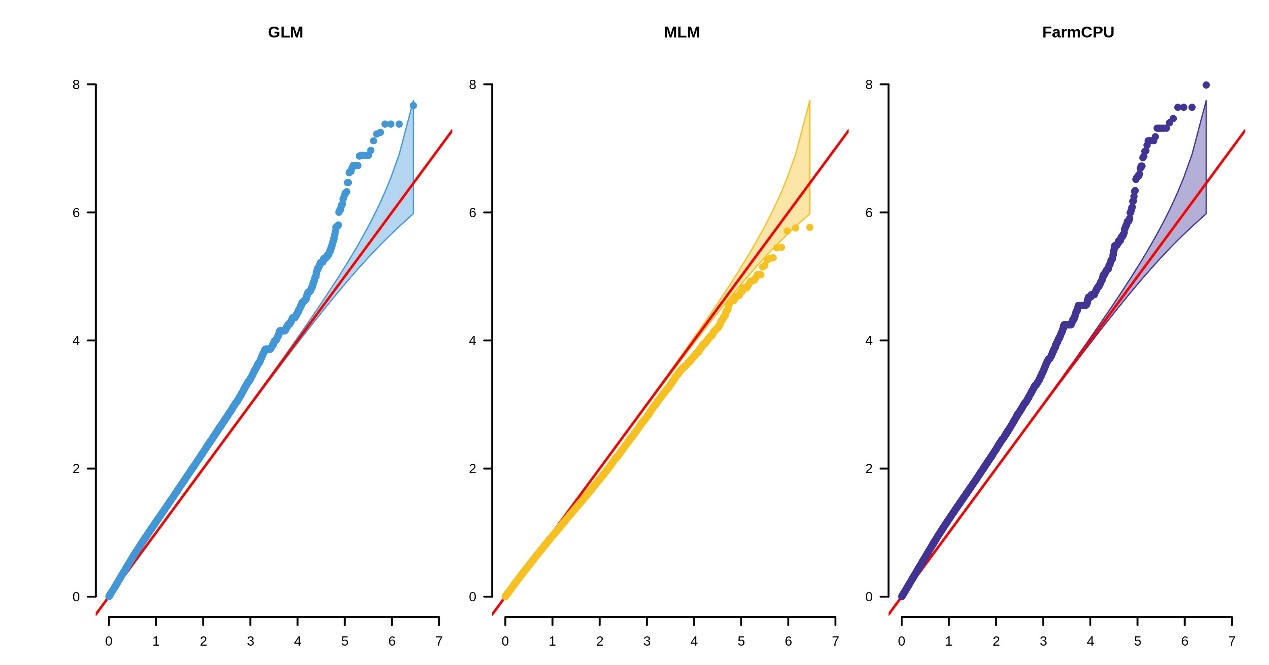


**Fig. S4** Quantile-quantile plots for the GWAS in *japonica* population using GLM, MLM and FarmCPU.


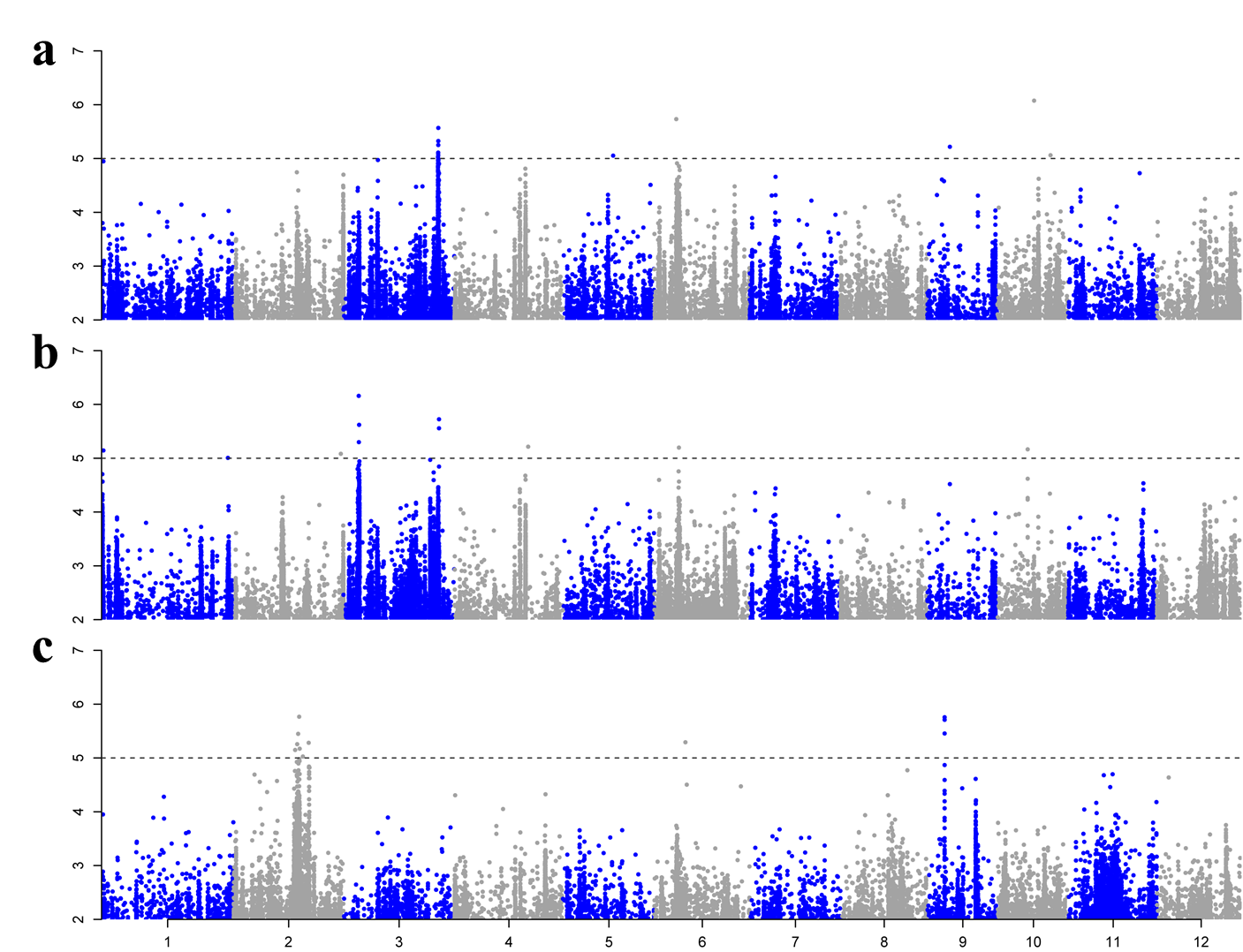


**Fig. S5** GWAS results of RBLS in different populations using MLM. Manhattan plots for the GWAS in full (**a**), *indica* (**b**), and *japonica* (**c**) using MLM. The red and black genes show known genes and candidate genes involving in the RBLS mentioned in the text, respectively. A dotted horizontal line for each figure indicates the significance threshold (*P* = 10^−5^).


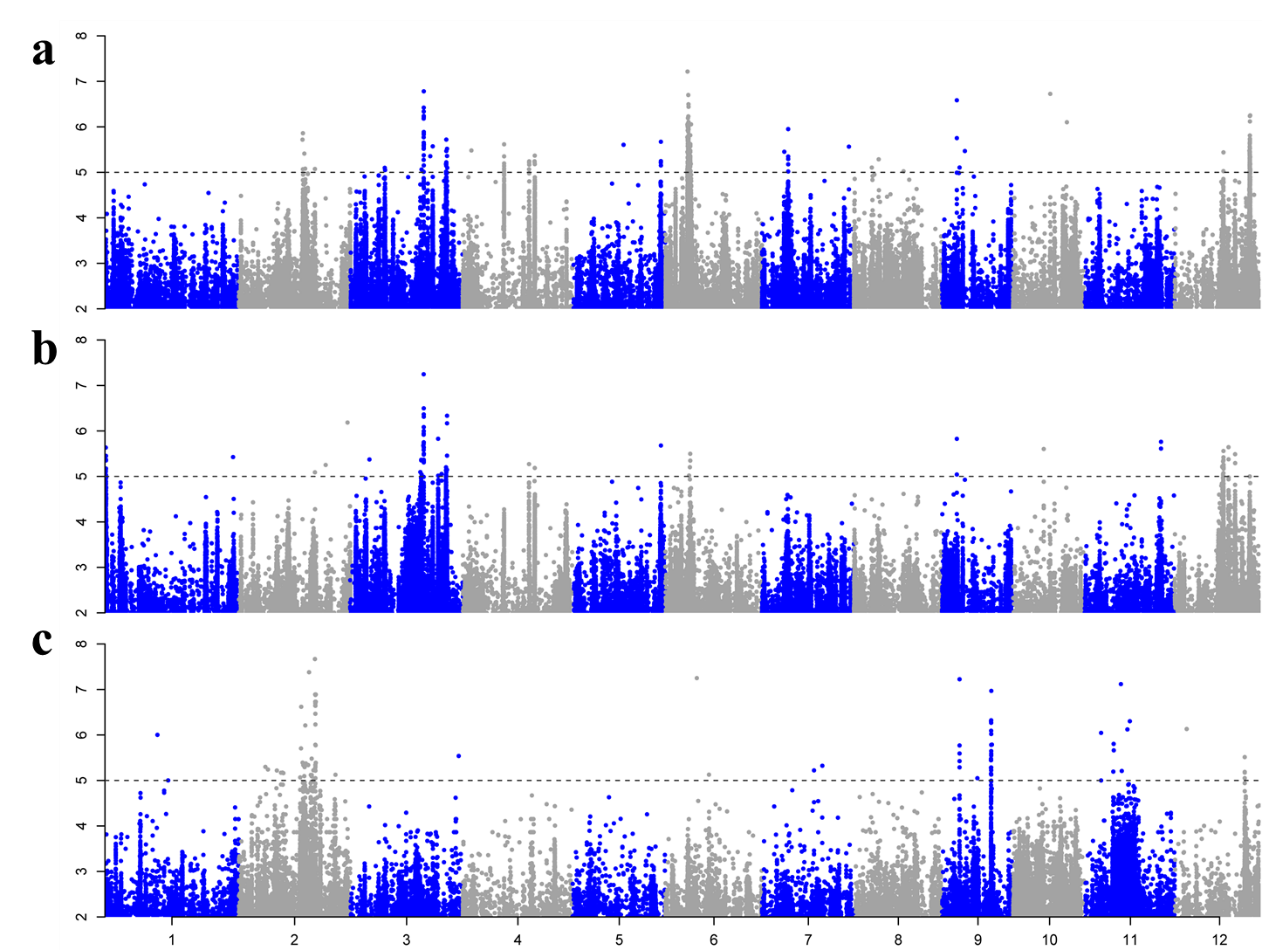


**Fig. S6** GWAS results of RBLS in different populations using GLM. Manhattan plots for the GWAS in full (a), *indica* (b), and *japonica* (c) using GLM. The red and black genes show known genes and candidate genes involving in the RBLS mentioned in the text, respectively. A dotted horizontal line for each figure indicates the significance threshold (*P* = 10^−5^).


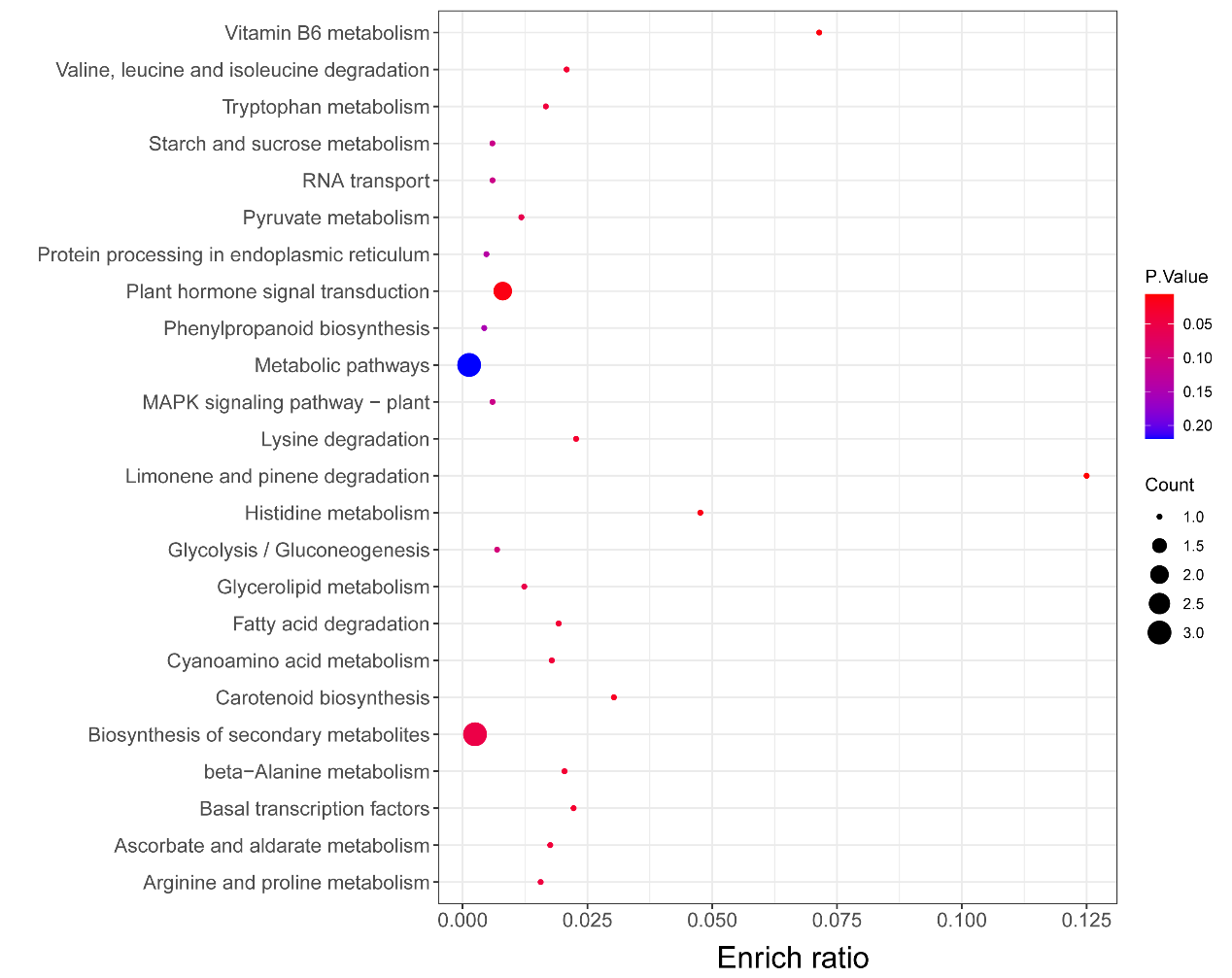


**Fig. S7** Pathway enrichment of the cloned genes for RBLS.


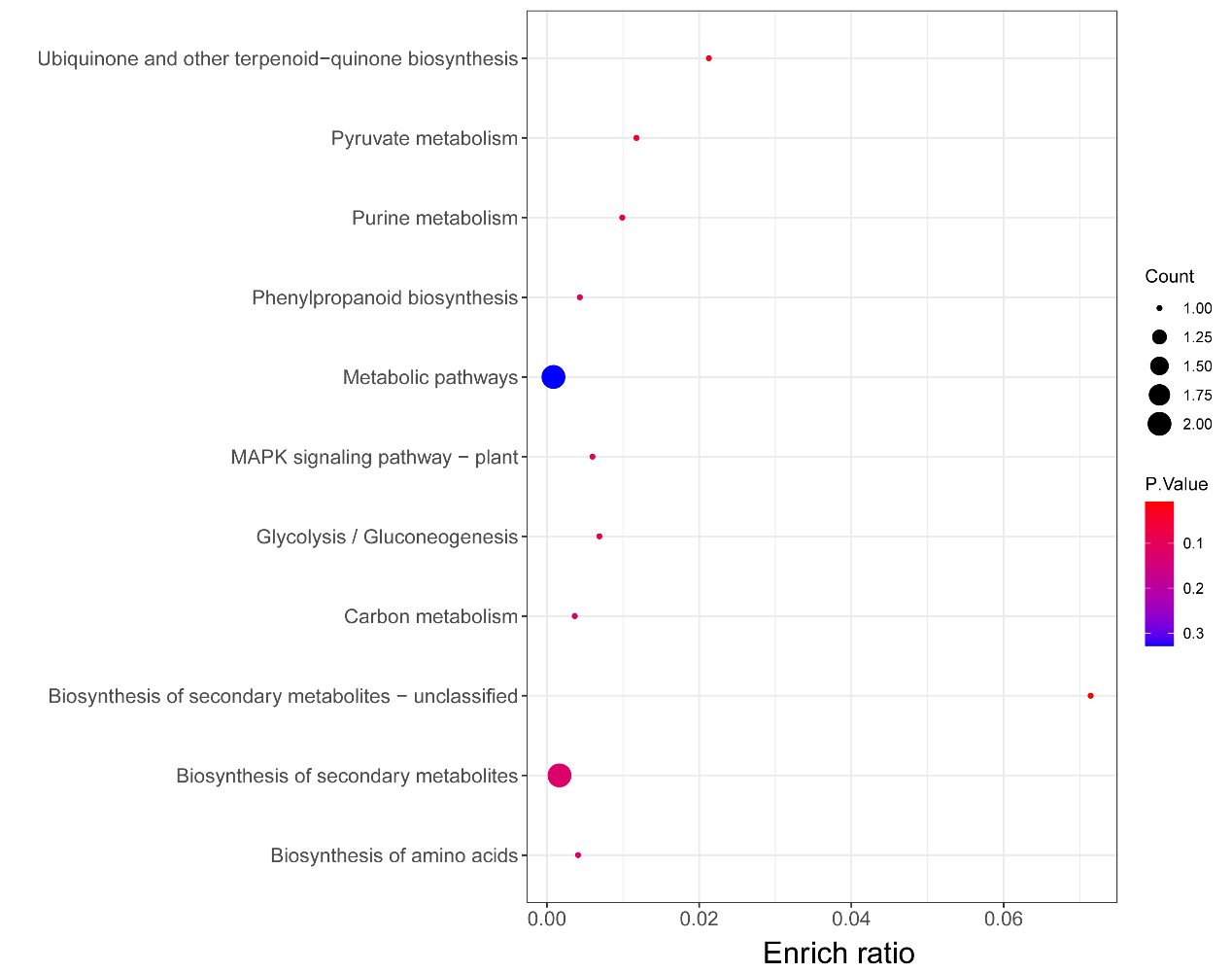


**Fig. S8** Pathway enrichment of the candidate genes for RBLS.
